# Photo-Detectors for Time of Flight Positron Emission Tomography (ToF-PET)

**DOI:** 10.3390/s101110484

**Published:** 2010-11-18

**Authors:** Virginia Ch. Spanoudaki, Craig S. Levin⋆

**Affiliations:** 1 Molecular Imaging Program at Stanford (MIPS) and Department of Radiology, Stanford University, 300 Pasteur Drive 94305, Stanford, CA 94305, USA; E-Mail: v.spanoudaki@stanford.edu; 2 Departments of Electrical Engineering and Physics, Stanford University, Stanford, USA

**Keywords:** positron emission tomography, time of flight, silicon photo-multipliers

## Abstract

We present the most recent advances in photo-detector design employed in time of flight positron emission tomography (ToF-PET). PET is a molecular imaging modality that collects pairs of coincident (temporally correlated) annihilation photons emitted from the patient body. The annihilation photon detector typically comprises a scintillation crystal coupled to a fast photo-detector. ToF information provides better localization of the annihilation event along the line formed by each detector pair, resulting in an overall improvement in signal to noise ratio (SNR) of the reconstructed image. Apart from the demand for high luminosity and fast decay time of the scintillation crystal, proper design and selection of the photo-detector and methods for arrival time pick-off are a prerequisite for achieving excellent time resolution required for ToF-PET. We review the two types of photo-detectors used in ToF-PET: photomultiplier tubes (PMTs) and silicon photo-multipliers (SiPMs) with a special focus on SiPMs.

## 1. Introduction

Recent advances in the field of medical imaging have greatly facilitated the transition from technologies used to accurately image structures inside the human body to technologies sensitive enough to provide functional and biological information at the cellular and the molecular level. Positron emission tomography (PET) is considered to be one of the most sensitive in-vivo molecular imaging modalities despite its significantly inferior spatial resolution compared to imaging modalities such as computed tomography (CT) and magnetic resonance imaging (MRI). The improvement of PET detector technology is an active field of research and efforts are focused on addressing the limits in spatial resolution and sensitivity achieved in PET.

### 1.1. Positron emission tomography (PET): Basic principles

**Figure 1.**
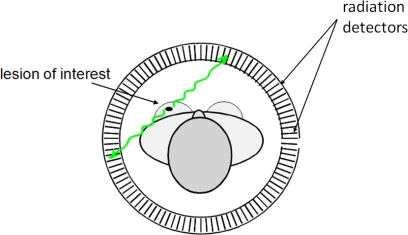
The basic principle of PET: a ring of detectors placed around the object detects photon pairs (green arrows) which are generated as a result of the annihilation of a positron emitted by the radio-pharmaceutical.

PET imaging is performed after the administration to the patient of a radio-tracer, namely a biomolecule labeled with a radioactive atom emitting positrons (*β*^+^ particles). The biomolecule is chosen such that it will preferentially accumulate in the area of interest resulting in high radio-tracer concentration in this region. A PET acquisition is based on the coincident detection of many pairs of simultaneous anti-parallel photons following the annihilation of the positron emitted by the radio-tracer [1]. The detection is performed by a number of detector element pairs which are placed around the imaged object, typically in a ring geometry, as shown in Figure 1. The incident annihilation photons will interact with these detectors which will fully or partially absorb the photon energy resulting in the generation of an electrical signal. This process will be henceforth referred to as a photon event. A PET acquisition will eventually result in a number of identified detector pairs that have simultaneously detected the two annihilation photons, or otherwise stated, the positron annihilation event. These pairs will be subsequently assigned a virtual line connecting the two detection points (line of response, LoR). For every stimulated detector pair the related positron annihilation is localized somewhere along the respective LoR through the patient with no further information about the exact point of positron annihilation along that line. Acquisition from detector pairs at various angular views (tomographic acquisition) followed by appropriate reconstruction algorithms allows for estimation of the tracer biodistribution within the imaged object with a finite spatial resolution and sensitivity.

PET detectors are required to have fast response due to the requirement for detection of simultaneous photon events. To date the detectors typically used in PET have a finite time resolution of a few nanoseconds (ns) allowing for detection of photon events within a predefined time window. The width of this window is usually chosen to be twice the time resolution of the PET detectors.

### 1.2. The time of flight (ToF) feature: Benefits and challenges

**Figure 2.**
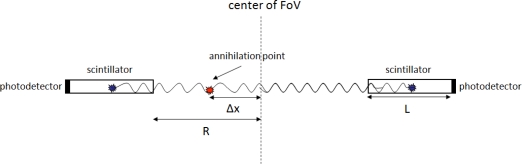
The ToF concept: the flight time difference Δt between the two detected photon events is in a first approximation related to the object position Δx along the line connecting the two detector elements through Equation 1.

Time of flight PET (ToF-PET) is an advance over traditional PET that exploits the time difference Δt in detection of the two photon events and correlates it to the position Δx of the annihilation point with respect to the center of the field of view (FoV) according to the formula (Figure 2) [2]:

(1) $$\Delta x=\frac{c\Delta t}{2}$$

Even though the concept of ToF PET and its implementation in PET system designs started in the early 1980’s [3], the development of new fast scintillation materials and the rapid advances in photo-detection technology have recently led to increased interest in the development of novel ToF-PET detector designs as well as to commercialization of the first ToF-PET clinical systems (Section 3).

**Figure 3.**
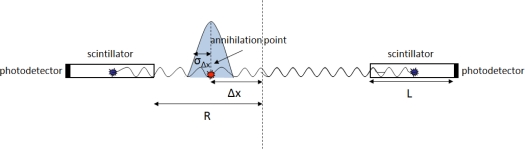
The limited detector time resolution associates the estimated position Δx with a variance $\sigma_{\Delta x}^{2}$.

Due to the limited time resolution of the PET detectors the registered time difference Δt is blurred by a variance $\sigma_{\Delta t}^{2}$ resulting in a corresponding blurring in the estimated position Δx by a variance $\sigma_{\Delta x}^{2}$ (Gaussian structure in Figure 3).

**Figure 4.**
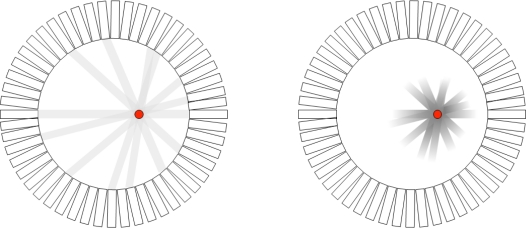
Illustration of the line back-projection process for the conventional PET system (left) and a ToF-PET system (right). Essentially more counts are placed at or near the correct position indicated by a red dot.

To date the time resolution of PET detectors is not adequate in order for ToF information to be used for exact localization of the annihilation point along the LoR. For example, a detector time resolution of 500 picoseconds (ps) will result in a position blurring of 7.5 cm. This blurring is an order of magnitude higher compared to the spatial resolution of several mm achieved by current clinical PET systems [4] and dominated by the scintillation crystal pixel size [5]. However, time resolution values of hundreds of ps that current detectors have can result in a considerable gain in signal-to-noise ratio (SNR) in the reconstructed image which further implies better lesion detectability, reduced radioactive dose received by the patient and/or reduced scan time [6–8]. An illustration of the difference between a standard PET and a ToF-PET acquisition is illustrated in Figure 4.

### 1.3. The concept of time resolution

We define the time resolution of a detector as the minimum time interval between two subsequent photon events in order for these to be recorded as separate events [9]. In PET, time resolution is typically measured for pairs of detectors that detect many annihilation photon events and is defined as the full width at half maximum (FWHM) of the distribution of time difference between the two detectors, over multiple events. Typical time resolutions achieved by modern PET systems lie within the 2–10 ns range. In the case of ToF-PET, however, such magnitudes are insufficient in order for ToF information to have a benefit over a conventional PET acquisition. The existing commercially available ToF-PET imaging systems can achieve time resolutions in the range of 500–900 ps allowing for a Gaussian weighted localization with a FWHM of 7.5–13.5 cm along each LoR.

The lowest limit in time resolution for a single photon event that can be achieved is theoretically described by a number of empirical formulas [10,11] and for a generic detector with finite signal-to-noise ratio and transit time jitter could be summarized as follows:

(2) $$\sigma_{\mathrm{time}}^{2}\approx {\left(\frac{\sigma_{\mathrm{noise}}}{\frac{\mathrm{dV}}{\mathrm{dt}}}\right)}^{2}+\sigma_{\mathrm{TTS}}^{2}$$

where $\sigma_{\mathrm{time}}^{2}$ is the estimated time variance, σ*_noise_* is the square root of noise variance superimposed on the detector signal, $\frac{\mathrm{dV}}{\mathrm{dt}}$ is the signal slope at the point of time estimation (time pick-off) and $\sigma_{\mathrm{TTS}}^{2}$ is the time variance due to the photo-detector transit time spread. From the above formula it can be understood that timing resolution is dependent on (1) the electronic and detector noise (as represented by σ*_noise_*), (2) the combination of detector signal gain and detector signal time response (as represented by $\frac{\mathrm{dV}}{\mathrm{dt}}$), (3) the triggering level at which the detector noise and slope are calculated and (4) the inherent time jitter of the photo-detector’s charge carriers (as represented by σ*_TTS_*) [12]. The desired time resolution requirements for ToF-PET aim for detector signals with low noise, high gain and fast time response. Basic error propagation theory dictates that for photon pair coincidence time resolution the right hand side of Equation 2 be multiplied by 2. A more detailed expansion of Equation 2 will eventually lead to the well known $\frac{1}{\sqrt{N}}$ law for the dependence of the time resolution (FWHM) on the number of generated photoelectrons N.

Even though there have been several studies in the past demonstrating the feasibility of ToF PET, only the past few years has it managed to gain popularity in both industry and research. Recent technological advances have led to the development of new scintillator materials and novel photo-detector architectures with improved performance properties that have significantly facilitated the rapid integration of ToF technology into complete imaging systems. In the following, we review the most important components of ToF-PET radiation detectors with emphasis in solid-state photo-detectors.

## 2. Radiation Detectors for Fast Timing Applications

**Figure 5.**
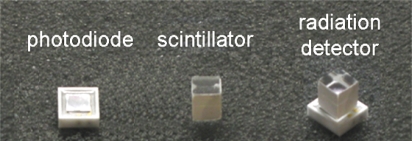
Photo of a radiation detector based on a scintillator/photo-detector combination.

Typically in PET the detection of incident radiation is achieved by a two-step conversion of the annihilation photon energy (1) to visible light in a scintillation material and (2) to electric charge in a photo-detector. Even though inevitable signal losses are associated with this two-step conversion, this type of detector is still considered to be a well understood, fast and efficient means of 511 keV photon detection. An example of a two-step radiation detector is shown in Figure 5.

### 2.1. The ideal ToF-PET detector

In order to extract as accurately as possible information about the flight time difference of the two annihilation photons, both components of the PET detector, namely the scintillator and the photo-detector, should have a fast, ideally instantaneous response. However, there are a number of factors which may considerably affect time resolution, such as the statistical nature of radiation detection, even in the case of very fast response. Both the detection of the annihilation photons in a scintillation crystal as well as the detection of optical photons in the photo-detector are subject to Poisson statistics which may affect the timing accuracy especially in case of a low scintillation light yield and/or a low photo-detector gain. Thus high light output in the scintillator and high gain in the photo-detector are key features in order to minimize statistical uncertainties.

An ideal ToF-PET radiation detector will combine the following features:

1. a scintillator material with high efficiency for absorption of the 511-keV annihilation photons
2. a scintillation process with instantaneous rise and decay times of the light pulse profile
3. a scintillator geometry with a low aspect ratio ( $=\frac{\mathrm{length}}{\mathrm{width}}$) which will minimize the optical photon travel path and thus light losses at the crystal interfaces, as well as transit time variances in the crystal
4. a photo-detector with high efficiency in the detection of the visible light produced by the scintillator
5. a photo-detector with high amplification gain and low noise
6. a photo-detector with instantaneous rise and decay time of the charge pulse profile and low charge transit time spread

**Figure 6.**
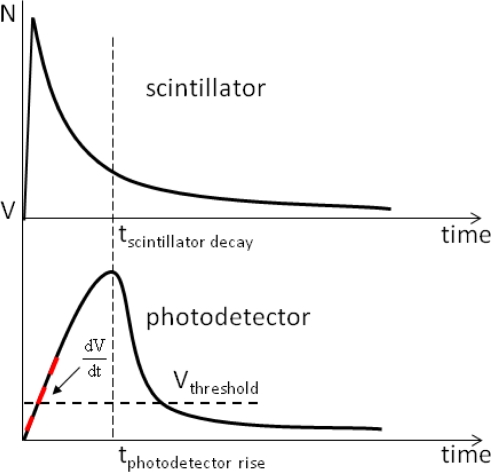
Illustration of typical response profiles of a scintillator (top, response to high energy photons) and of a photo-detector (bottom, response to scintillation light from top plot). The red dashed line indicates the signal slope ( $\frac{\mathrm{dV}}{\mathrm{dt}}$) at the triggering threshold (*V_threshold_*), which together with the noise superimposed on the signal determine the lower bound on timing resolution for a single detector signal according to Equation 2.

Typical response profiles of a scintillator and a photo-detector are shown in Figure 6. Extraction of time information is performed on the electrical pulse generated by the photo-detector. The properties of this pulse will be affected by both the scintillator and the photo-detector.

### 2.2. Scintillation crystals

Table 1 summarizes the basic features of fast scintillators that have been considered in ToF-PET, either in research or in industry. As already mentioned in the description of an ideal radiation detector, a scintillator with high photon emission rate (high photon yield at a fast decay time) and high stopping power (as represented by the effective atomic number and the density) is the desired combination for ToF-PET. However additional features such as the refractive index and the peak emission wavelength will assure compatibility with the photo-detector and thus efficient transfer of the optical photon flux from the scintillator to the photo-detector. Even though LSO or LYSO is acknowledged as the option of choice for fast scintillation counters, an even faster and more luminous scintillation material, LaBr_3_ has recently attracted interest with promising results [19–21] despite its hygroscopic properties and lower stopping power compared to LSO. Recently, the doping of the latter (LSO) with Ca in addition to Ce dopants proves to improve the timing performance of the scintillator.

From the materials listed in Table 1, BaF_2_ and plastic scintillators are not preferred options for ToF-PET despite their excellent time response. Plastic scintillators suffer from low stopping power and optical photon yield, factors that will significant compromise PET overall imaging performance. The inorganic material BaF_2_ has been studied in the past as a candidate for ToF [22] with promising results in terms of time resolution reaching values below 300 ps [23]. However, the stopping power and light output of this material is significantly lower compared to the other inorganic scintillators of Table 1 and, in addition, the peak emission wavelength of the fast decay component of BaF_2_ may restrict the available choices of photo-detectors with sufficient light detection efficiency at this part of the electromagnetic spectrum.

### 2.3. Photo-detectors

Even with an ideal scintillator yielding high optical photon rate, a lower bound on the time resolution that can be achieved is imposed by the photo-detector performance. Low quantum efficiency (ratio of number of emitted charge to the number of incident optical photons absorbed in the photosensitive area, QE) of the photo-detector at the peak emission wavelength of the scintillator will result in an effective reduction of the amount of scintillation light detected. In addition low gain, gain variations or noise in the photo-detector response will increase statistical variations in the extraction of any type of information from the generated electrical signal. Table 2 summarizes the basic performance features of the three types of photo-detectors widely used in commercial and research PET systems [24,25].

From the photo-detectors listed in Table 2, proportional mode avalanche photo-diodes (APDs) are not preferred for use in the ToF application due to their significantly slower response time. In addition, their low multiplication gain results in small output signals requiring further amplification prior to processing. In the following, two types of photo-detectors will be reviewed, the PMT and the SiPM, with special focus on the latter.

#### The photomultiplier tube (PMT)

**Figure 7.**
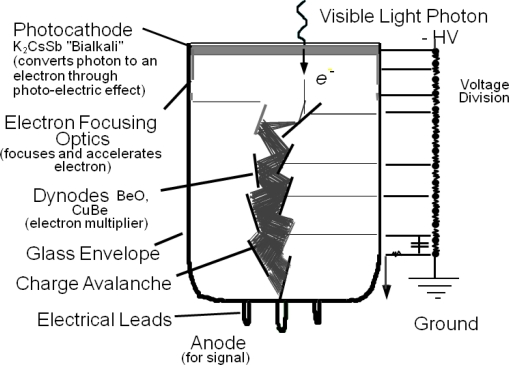
Schematic diagram and principle of operation of a PMT. For illustration purposes only a few HV connections to the dynodes are shown.

The photomultiplier tube (PMT) is a photo-detector type commonly used for scintillator readout in numerous applications including medical imaging. In ToF-PET, PMTs are still considered to be the gold standard for sub-nanosecond time resolution [12,26]. Their high gain combined with a low excess noise factor lead to minimal deviation from Poisson statistics as well as reduced statistical uncertainty in the generated charge signal [9]. The basic component of a PMT is a vacuum tube consisting of a photo-cathode, several electrodes called dynodes and an anode. The photocathode is a photo-sensitive electrode that emits charge (electrons) for a number of incident optical photons absorbed with a given efficiency (QE defined above). Between the cathode and the anode a bias voltage in the kV range is applied to facilitate the generated electron transport and amplification from the cathode to the anode. Under the influence of a high potential the generated electrons from the photocathode drift and successively encounter the dynode stages. At every dynode stage the incident electrons have gained sufficient energy to create secondary electron emissions from collisions with the dynode, thus resulting in a large electron cloud at the anode (Figure 7). As can be seen in Table 2, one advantage of PMTs over semiconductor photo-detectors is their lower sensitivity in temperature variations which in most cases implies stable detector operation over a wide range of temperatures. However, their relatively bulky size and the requirement for power supplies which can provide thousands of Volts make PMTs a less attractive option for their use in high packing fraction, high channel density PET radiation detectors.

#### The Silicon photomultiplier (SiPM)

**Figure 8.**
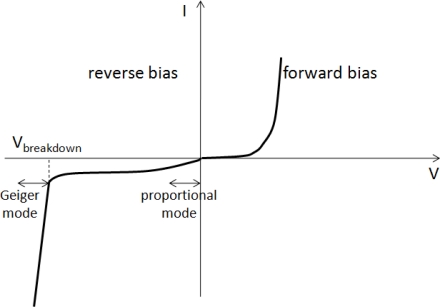
The characteristic (I-V) curve of a typical p-n diode. Avalanche photo-diodes (APDs) operate at a reversed applied bias. Proportional APDs operate at the regime of bias voltages well below breakdown where the amount of produced charge is proportional to the number of absorbed optical photons. SiPMs and their individual components (G-APDs or micro-cells) operate at bias voltages above breakdown (V*_breakdown_*) where the avalanche process becomes excessive. At this operation mode (Geiger-mode) the amount of charge produced from each micro-cell is standardized and the total amount of charge produced from all micro-cells is proportional to their number.

**Figure 9.**
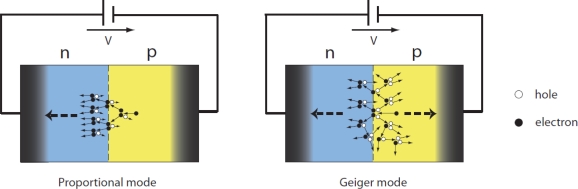
The difference between a proportional APD and a G-APD. Illustration is courtesy of Dr. A. Nepomuk Otte, University of Santa Cruz. Adopted with permission [33].

The silicon photomultiplier (SiPM) has recently been introduced to address the shortcomings of both PMTs and APDs summarized in Table 2 [25,27–32]. These devices consist of an array of microscopic, parallel connected avalanche photo-diodes which operate in limited Geiger-mode (alternatively known as G-APDs or SiPM micro-cells). In this mode of operation the micro-cells are biased above breakdown resulting in a cumulative avalanche breakdown within the diode depletion region and thus in an excessive current every time they encounter a (visible) photon stimulus (Figure 8). The difference between the proportional and the Geiger mode of APD operation is illustrated in Figure 9. As shown in Figure 9 in the case of a proportional-mode APD the electron carriers are primarily involved in the avalanche process, while in the case of a Geiger-mode APD both charge carriers (namely electrons and holes) are able to trigger an avalanche.

**Figure 10.**
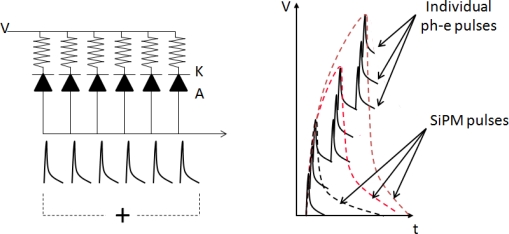
Left: schematic of the equivalent electrical circuit of a SiPM. Only 6 micro-cells, each represented by a diode symbol, are shown. Right: illustration of the signal formation in a SiPM. The pile-up of the individual micro-cell pulses is achieved by means of summing via a common readout line.

In order for the large amount of current not to destroy the diode, an appropriate quenching circuit is integrated to each micro-cell as shown in Figure 10. Typically, a resistor of several hundreds of kOhm (quenching resistor) is connected in series with each micro-cell to quench the avalanche (passive quenching). Every time a large current flows trough it, it will result in a voltage drop across the micro-cell terminals. This voltage drop is large enough to lower the micro-cell applied bias below the breakdown limit, resulting in interruption of the avalanche process. Due to the inherent micro-cell capacitance, as well as the parasitic capacitance of the overall summing network in a SiPM, each micro-cell needs a characteristic amount of time to recover after the bias voltage is restored to values above breakdown. This characteristic time is called recovery time and its magnitude is typically equal to the product of the value of the quenching resistor and the value of the overall diode capacitance.

Even though passive quenching is relatively simple to implement and most of commercially available SiPMs employ such circuits, there are specific performance limitations associated with it. The pulse shape depends on the values of the passive elements used in the quenching circuitry and may thus be subject to variations depending on how these values are influenced *i.e.*, by temperature. In addition SiPMs with passive quenching are typically characterized by long recovery times which can subsequently impact their count rate performance and dynamic range [34].

**Figure 11.**
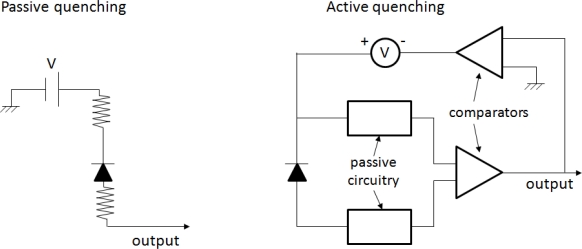
Generic schematics of a passive (left) and an active (right) quenching circuit employed at the micro-cell level (the micro-cell is represented by the diode symbol).

In order to address the above shortcomings of passive quenching, G-APDs can alternatively be quenched using an active quenching circuit (Figure 11).

A generic active circuit typically consists of a comparator-based feedback circuit with a sensing and a quenching network. Whenever the rise of the G-APD output pulse is sensed, indicating the rise of an avalanche, a feedback pulse is produced and forces the G-APD to quench the avalanche by means of a controlled voltage source. This source is also configured to reset the G-APD back to an active state by restoring the proper bias across its terminals. Both the quenching and the reset are performed in a highly controlled way allowing thus for fast recovery of the SiPM micro-cells [35]. For applications where high count rate capability is a prerequisite, SiPMs with active quenching are preferred.

Even though the most acknowledged advantage of actively quenched SiPMs is their enhanced count rate performance, they may also demonstrate an effective enhancement in PDE owing to the fact that each micro-cell can be triggered multiple times, within the duration of the light stimulus, due to their fast recovery (factor RT becomes larger in Equation 4). On the other hand, it is difficult to predict quantitatively the actual PDE enhancement because of the limitations in the SiPM fill factor due to the active quenching circuitry (factor FF becomes smaller in Equation 4).

**Figure 12.**
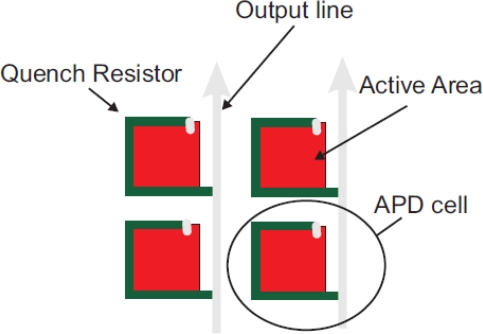
Generic schematic of the micro-cell configuration within a passively quenched SiPM. The quenching resistor as well as the Al readout trace are depicted. Illustration is courtesy of Dr. A. Nepomuk Otte, University of Santa Cruz. Adopted with permission [33].

In both the aforementioned quenching modes, every micro-cell acts as a binary device which can provide information about the detection of a light stimulus but no information about its intensity. Proportionality between the intensity of the incident light stimulus and the output charge of a SiPM is achieved by summing the signals of individual micro-cells through a common readout line, provided that the incident photon flux is low enough in order for each micro-cell to respond to a single visible photon (Figures 10 and 12). The SiPM combines appealing detector features of both the PMT and the APD for ToF-PET imaging:

1. Small size which enables detectors with high packing fraction for enhanced sampling and photon sensitivity. In addition small sensitive area reduces device capacitance and dark current.
2. High gain which eliminates the need for pre-amplification.
3. Single electron pulses from each micro-cell with a very well defined pulse shape which can be exploited for optimum timing.

One of the most important limitations in a SiPM design is the trade-off between dynamic range and light detection efficiency. A sufficient number of available cells are needed for a SiPM to respond linearly to the incident scintillation light stimulus according to the theoretical formula:

(3) $$N_{\mathrm{fired}\;\mathrm{cells}}=N_{\mathrm{cells}}\cdot (1-e^{-\frac{N_{\mathrm{incident}}\cdot \mathrm{PDE}}{N_{\mathrm{cells}}}})$$

where N*_cells_* is the number of micro-cells contained in a SiPM sensitive area, N*_fired cells_* is the number of stimulated micro-cells, N*_incident_* is the number of optical photons incident to the SiPM area and PDE is the SiPM photon detection efficiency defined empirically by the following formula:

(4) PDE=QE⋅FF⋅GP⋅RT

where FF is the geometric fill factor defined as the ratio of active to total area, GP is the probability for Geiger discharge in any micro-cell and RT is a factor related to the recovery time of every micro-cell. Equation 3 is an approximation which does not take into account crosstalk and after-pulse events (discussed below). In addition it assumes an infinitesimally long recovery time of each micro-cell and and a low incident photon flux limited to less than $\frac{1\;\mathrm{photon}}{\mathrm{microcell}\cdot \mathrm{recovery}\;\mathrm{time}}$.

**Figure 13.**
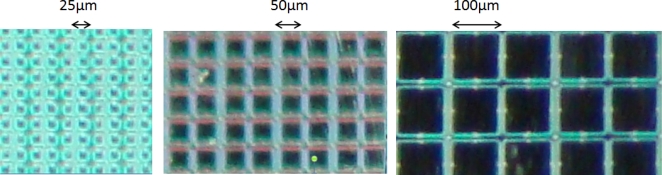
Microscope captures of sensitive areas of SiPMs with micro-cell size of 25 *μ*m (left), 50 *μ*m (middle) and 100 *μ*m (right). The fill factor increases with increasing micro-cell size, while the dynamic range (number of available micro-cells within a given area) decreases.

Furthermore in a given SiPM area with a fixed dead area (area occupied by the read-out traces) around each micro-cell, a larger number of micro-cells would imply a smaller micro-cell sensitive area. This decreased micro-cell area decreases the light PDE of each cell and thus, the overall detection efficiency of the device is degraded (Figure 13).

**Figure 14.**
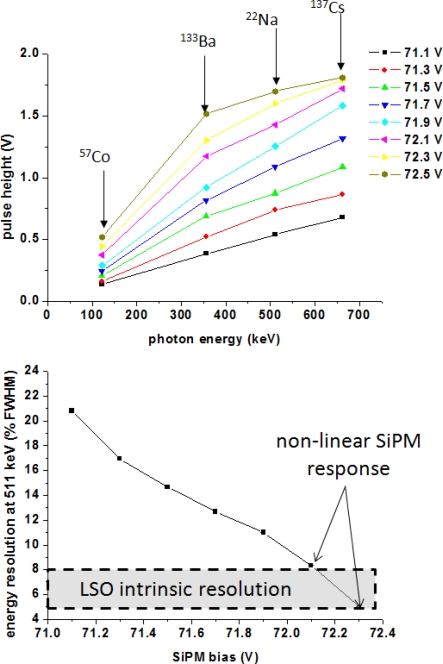
Effects of non-linear SiPM operation on identification of different photon energies (top) and energy resolution (bottom) as a function of bias voltage. Data taken with a SiPM with a 3 × 3 mm^2^ cross-sectional area from Hamamatsu (50 *μ*m micro-cell size). The scintillator used was a 3 × 3 × 5 mm^3^ LSO crystal element.

The plots of Figure 14 indicate severe non-linear effects and effects on the energy resolving power of scintillation readout from SiPMs as a function of their applied bias voltage. Above a given value of the applied voltage the measured SiPM response to radiation quanta of different energies is no longer linear (Figure 14, top). An increasing bias voltage leads to increased SiPM gain and PDE, initially resulting in energy resolution improvements. For excessive bias increase, the PDE is high enough to effectively lower the SiPM dynamic range (because the number of optical photons which can trigger an avalanche is no longer related linearly to the number of available micro-cells). In this case, the measured energy resolution values appear to be overestimated, namely below the theoretical limits imposed by the intrinsic resolution of the scintillator (Figure 14, bottom).

**Figure 15.**
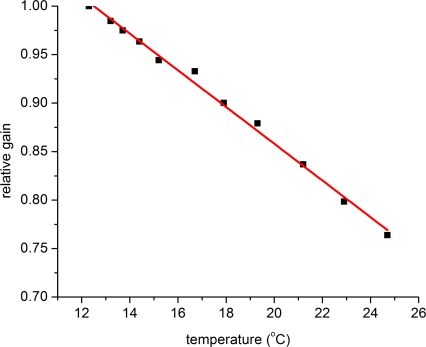
Dependence of SiPM gain on temperature. Data taken with a SiPM with a 1 × 1 mm^2^ cross-sectional area from Hamamatsu (50 *μ*m micro-cell size). All the temperature measurements were performed at a constant SiPM bias.

As in any semiconductor, temperature sensitivity of SiPMs is still a major issue affecting the detector’s performance [36,37]. Temperature coefficients varying from 2 to $8\frac{\%}{{}^{\circ}C}$ have been measured. As shown in Figure 15, the detector gain is decreased with increasing temperature owing to quenching of a larger part of the avalanche carriers’ energy due to collisions with the crystal lattice vibrations (phonons). In addition, for the majority of SiPMs employing passive quenching, the value of the quenching resistor demonstrates a temperature dependence that may lead to variations in the SiPM pulse shape with further implications on recovery time and timing performance of the devices [38].

**Figure 16.**
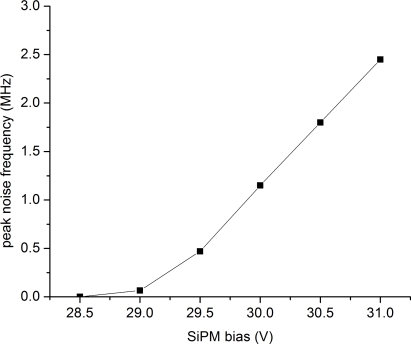
Dependence of dark counts on SiPM bias. Data taken with a SiPM with a 3×3 mm^2^ cross-sectional area from SensL (50 *μ*m micro-cell size).

SiPMs are characterized by high dark count rates in the kHz to MHz range. Dark counts are signals induced in the individual micro-cells by a thermal stimulus instead of a light signal. Thermal stimulation is frequent in SiPM micro-cells and can result in an increased signal noise level thus affecting the timing performance of the devices (Equation 2). Figure 16 shows the dependence of the maximum registered dark count frequency on SiPM applied bias. At a given applied bias, multiple dark count rates may exist depending on the triggering threshold at which these rates are measured. This effect is attributed to the optical crosstalk (described below) apparent between different micro-cells of a SiPM, resulting in simultaneous signal generation from multiple micro-cells. The triggering threshold for the measurements shown in Figure 16 is adjusted depending on the applied bias, so that the maximum dark count rate typically from a single micro-cell (denoted as ’peak noise frequency’ in the y-axis of Figure 16) is registered.

An effective increase in the observed SiPM dark count rate is also attributed to after-pulse effects. After-pulses are belated micro-cell excitations following the initial micro-cell excitation due to late release of avalanche charge carriers trapped in defects within the diode sensitive area (depletion region). This phenomenon effectively results in small signals piled up in the tail of the actual SiPM output [39].

The Geiger discharge followed by the excessive avalanche process results in inherent light emission from the micro-cells themselves [40]. The SiPM architecture is such that crosstalk due to distribution of this light between adjacent micro-cells may occur. This process is known as optical crosstalk and leads to falsely stimulated micro-cells and furthermore to an effective decrease of the actual SiPM dynamic range. Optical crosstalk may be further facilitated by optical media such as the SiPM entrance window (typically coated by epoxy or resin) or the scintillation crystal itself.

The aforementioned limitations in SiPM performance are being addressed by a number of specialized designs:

- Back-illuminated SiPMs for enhanced PDE [41]
- SiPMs with an enhanced p-on-n structure for sensitivity in blue light [42]
- SiPMs with quenching resistors in the bulk for elimination of the polysilicon resistor [43]
- SiPMs with dual p-n structure for cross-talk elimination [44]
- SiPMs with intra-cell trenches for cross-talk elimination [44]

**Figure 17.**
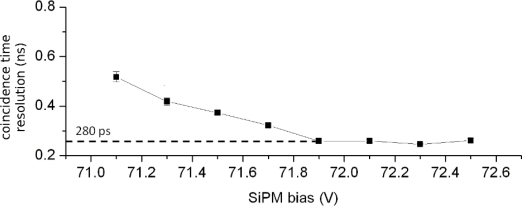
Dependence of coincidence time resolution on SiPM bias (measurement performed for a pair of LSO-SiPM detectors). Data taken with two SiPMs with a 3 × 3 mm^2^ cross-sectional area from Hamamatsu (50 *μ*m micro-cell size). The LSO crystal size was 3 × 3 × 5 mm^3^. The time resolution value at the optimum bias setting is ∼280 ps (FWHM).

It is expected that in the near future these shortcomings will be eliminated to a large extent. However, despite the aforementioned challenges and limitations, scintillator readout by SiPMs shows great promise for fast timing applications, such as ToF-PET (Figure 17):

- intrinsic time resolution values at the single photoelectron level as low as 70 ps have been reported [45]
- LaBr_3_ readout with SiPMs demonstrates time resolution in the range of 100 ps [20]
- LYSO readout with SiPMs demonstrates time resolution in the range of 300–200 ps [29]

## 3. ToF-PET State of the Art Systems

A number of ToF-PET scanners are currently commercially available. Even though the current generation of ToF-PET systems is based on PMTs, it is expected that in future systems the PET detectors will be SiPM-based in order to utilize compact detector designs while achieving comparable or, potentially, superior time resolution.

- Philips TF PET/CT. The basic detector component of the system consists of arrays of LYSO pixels (4 × 4 × 22 mm^3^ pixel size) read out by hexagonal arrays of PMTs. The achieved time resolution of the system varies between 600 and 900 ps depending on the photon event count rate [46].
- Siemens Biograph TruePoint PET/CT. The detectors of this system are arrays of LSO crystal pixels (4×4×20 mm^3^ pixel size) read out by arrays of PMTs and can achieve 500 ps time resolution [47].
- GE Discovery 690. This ToF system also employs arrays of LYSO crystal pixels (pixel size is 4.25 × 6.3 × 25 mm^3^) read out by PMTs. The achieved time resolution is 600 ps [48].

## 4. Future of ToF-PET Photo-Detector Technology

### 4.1. Digital SiPMs

A novel technological advance in the field of semiconductor photo-detectors has been recently developed by Philips and involves the integration within the SiPM sensitive area of basic processing electronics thus eliminating the need for external processing electronics [49,50]. Each micro-cell of the array is connected to an integrated counter (for extraction of energy information) and an integrated time to digital converter (TDC, for extraction of time information). This alternative SiPM design is known as ’digital SiPM’ and time resolutions as low as 150 ps (FWHM) with LYSO readout have been reported.

The development of fully digital photon detection technologies based on single photon avalanche photo-diodes has been reported by other groups [51] even though these technologies have not been tested for annihilation radiation detection. The so-called single-photon synchronous detectors (SPSDs) are able to measure both intensity and phase of the incident light which can be exploited to compute the position of a target based on the phase difference of the light incident to the target and the light reflected by the target and detected by the SPSD.

Apart from the obvious advantage of simplified external signal processing characterizing the digital implementation of single photon detectors, there are some potential disadvantages associated with this technology, which include an increase in power consumption, as well as reduced fill factor due to the space required for the additional electronic circuits. In addition, since the analog form of the generated signal is not available, there may be some lack of useful information content, mostly associated with pulse shape variations.

### 4.2. Discrete amplification photodiodes

Another SiPM design that aims to address both the limited PDE and the recovery time issues of passive quenching is employed by discrete amplification photo-diodes (DAPDs) [52,53]. The internal architecture of these devices is fundamentally different from the micro-cell array concept of traditional SiPMs. The charge produced from a single photosensitive area is distributed to a number of discrete amplifiers which are integrated in the diode’s bulk. Each charge packet will be subject to individual amplification and the output signal will be the sum of the amplified charge packets. Due to the absence of long traces surrounding each micro-cell, DAPDs have very low capacitance which results in short recovery times.

## Figures and Tables

**Table 1.** Comparative table of fast scintillators which can be considered for ToF-PET [9,13–18].

	LSO(Ce)	LSO(Ce, Ca)	LYSO(Ce)	LaBr_3_	BaF_2_	Plastic
Density ( $\frac{g}{{\mathrm{cm}}^{3}}$)	7.40	similar to LSO	similar to LSO	5.29	4.89	1.03
Effective atomic number (Z)	66	similar to LSO	similar to LSO	46	54	12
Rise time (ns)	≤ 0:5	similar to LSO	similar to LSO	0.2–0.5	∼0.03	0.7–1
Decay time (ns)	40	31@0.4% Ca	similar to LSO	15–26	0.8/620	2–5
Photon yield/keV	20–30	∼35	similar to LSO	63	1.8/10	10
Refractive index (n)	1.82	1.82	1.82	1.90	1.56	1.58
Hygroscopic	No	No	No	Yes	Slightly	No
Peak emission wavelength (nm)	420	420	similar to LSO	380	220/310	Various

**Table 2.** Comparative table of three types of photo-detectors typically used in PET: PMT, APD and SiPM.

	PMT	APD	SiPM
Gain	10^6^	50–1,000	∼106
rise time (ns)	∼1	∼5	∼1
QE @ 420 nm (%)	∼25	∼70	∼25–75 (PDE)
Bias (V)	>1,000	300–1,000	30–80
Temperature sensitivity ( $\frac{\%}{{}^{\circ}C}$)	<1	∼3	1–8
Magnetic field sensitivity	yes	no	no
Sensitive area	cm^2^	mm^2^	mm^2^
Price/channel ($)	>200	∼100	∼50

## References

[1] Cherry S, Sorenson J, Phelps M (2003). Physics in Nuclear Medicine.

[2] Budinger T (1983). Time-of-flight positron emission tomography-status relative to conventional PET. J. Nucl. Med.

[3] Mullani N, Ficke D, Hartz R, Markham J, Wong G (1981). System design of fast PET scanners utilizing time-of-flight. IEEE Trans. Nucl. Sci.

[4] Moses W (2007). Recent Advances and Future Advances in Time-of-Flight PET. Nucl. Instr. Meth. Phys. Res. A.

[5] Levin C, Hoffman E (1999). Calculation of positron range and its effect on the fundamental limit of positron emission tomography system spatial resolution. Phys. Med. Biol.

[6] Karp J, Surti S, Daube-Witherspoon M, Muehllehner G (2008). Benefit of time-of-flight in PET: Experimental and clinical results. J. Nucl. Med.

[7] Surti S, Karp J, Popescu L, Daube-Witherspoon ME, Werner M (2006). Investigation of Time-of-Flight Benefit for fully 3D PET. IEEE Trans. Med. Imaging.

[8] Surti S, Karp J (2009). Experimental evaluation of a simple lesion detection task with time-of-flight. Phys. Med. Biol.

[9] Knoll G (2001). Radiation Detection and Measurement.

[10] Paulus T (1982). Principles and Applications of Timing Spectroscopy. Application Note AN-42, EG&E.

[11] Bell R (1966). Comparison of leading-edge and crossover timing in coincidence measurements. Nucl. Instr. Meth. Phys. Res. A.

[12] Szczesniak T, Moszynski M, Swiderski L, Nassalski A, Lavoute P, Kapusta M (2009). Fast Photomultipliers for TOF PET. IEEE Trans. Nucl. Sci.

[13] Derenzo S, Weber M, Moses W, Dujardin C (2000). Measurements of the Intrinsic Rise Times of Common Inorganic Scintillators. IEEE Trans. Nucl. Sci.

[14] Spurrier M, Szupryczynski P, Rothfuss H, Yang K, Carey AA, Melcher CL (2008). The effect of co-doping in the growth stability and scintillation properties of lutetium oxyorthosilicate. J. Cryst. Growth.

[15] Szczesniak T, Moszynski M, Syntfeld-Kazuch A, Swiderski L, Koschan MAS, Melcher CL (2010). Timing resolution and decay time of LSO crystals co-doped with calcium. IEEE Trans. Nucl. Sci.

[16] Syntfeld-Kazuch A, Moszynski M, Swiderski L, Szczesniak T, Nassalski A, Melcher CL, Spurrier MA, Goliszek B, Kaminski P, Nowaczyk M (2009). Energy Resolution of Calcium Co-doped LSO:Ce Scintillators. IEEE Trans. Nucl. Sci.

[17] van Loef E, Dorenbos P, van Eijk C, Krämer K, Güdel HU (2001). High-energy-resolution scintillator: Ce^3+^ activated LaBr_3_. Appl. Phys. Lett.

[18] Saint Gobain http://www.detectors.saint-gobain.com.

[19] Kuhn A, Surti S, Karp J, Raby PS, Shah KS, Perkins AE, Muehllehner G (2004). Design of a lanthanum bromide detector for time-of-flight PET. IEEE Trans. Nucl. Sci.

[20] Schaart D, Seifert S, Vinke R, van Dam HT, Dendooven P, Löhner H, Beekman FJ (2010). LaBr_3_:Ce and SiPMs: achieving 100 ps coincidence resolving time. Phys. Med. Biol.

[21] Daube-Witherspoon M, Surti S, Perkins A, Kyba CCM, Wiener R, Kulp R, Karp JS (2010). The imaging performance of a LaBr_3_-based PET scanner. Phys. Med. Biol.

[22] Laval M, Moszynski M, Allemand R, Cormoreche E, Guinet P, Ordu R, Vacher J (1983). Barium Fluoride - Inorganic scintillator for subnanosecond timing. Nucl. Instr. Meth. Phys. Res. A.

[23] Ziegler S, Ostertag H, Kuebler W, Lorenz WJ, Otten EW (1990). Effects of scintillation collection on the time resolution of a time-of-flight detector for annihilation quanta. IEEE Trans. Nucl. Sci.

[24] Wernick M, Aarsvold J (2004). Emission Tomography: The fundamentals of PET and SPECT.

[25] Renker D (2006). Geiger-mode avalanche photodiodes, history, properties and problems. Nucl. Instr. Meth. Phys. Res. A.

[26] Moszynski M, Kapusta M, Nassalski A, Szczesniak T, Wolski D, Eriksson L, Melcher CL (2006). New Prospects for Time-of-Flight PET With LSO Scintillators. IEEE Trans. Nucl. Sci.

[27] Renker D (2007). New trends on photodetectors. Nucl. Instr. Meth. Phys. Res. A.

[28] Nassalski A, Moszynski M, Syntfeld-Kazuch A, Szczesniak T, Swiderski L, Wolski D, Batsch T, Baszak J (2010). Multi Pixel Photon Counters (MPPC) as an Alternative to APD in PET Applications. IEEE Trans. Nucl. Sci.

[29] Kim C, Wang GC, Dolinsky S (2009). Multi-Pixel Photon Counters for TOF PET Detector and Its Challenges. IEEE Trans. Nucl. Sci.

[30] Mazillo M, Condorelli G, Sanfilippo D, Valvo G, Carbone B, Fallica G, Billota S, Belluso M, Bonanno G, Cosentino L, Pappalardo A, Finocchiaro P (2009). Silicon Photomultiplier Technology at STMicroelectronics. IEEE Trans. Nucl. Sci.

[31] Piemonte C, Battiston R, Boscardin M, Dalla Betta G-F, Del Guerra A, DInu N, Pozza A, Zorzi N (2007). Characterization of the First Prototypes of Silicon Photomultiplier Fabricated at ITC-irst. IEEE Trans. Nucl. Sci.

[32] Stewart A, Saveliev V, Bellis S, Herbert DJ, Hughes PJ, Jackson JC (2008). Performance of 1-mm^2^ Silicon Photomultiplier. IEEE J. Quantum Electron.

[33] Otte A (2007). Observation of VHE γ-Rays from the Vicinity of Magnetized Neutron Stars and Development of New Photon-Detectors for Future Ground based γ-Ray Detectors.

[34] Spanoudaki V, Mann A, Otte A, Konorov I, Torres-Espallardo I, McElroy DP, Ziegler SI (2007). Use of single photon counting detector arrays in combined PET/MR: Characterization of LYSO-SiPM detector modules and comparison with a LSO-APD detector. J Instrum.

[35] Cova S, Ghioni M, Lacaita A, Samori C, Zappa F (1996). Avalanche photodiodes and quenching circuits for single-photon detection. Applied Opt.

[36] Sze S (1981). Physics of semiconductor devices.

[37] Petasecca M, Alpat B, Ambrossi G, Azzarello P, Battiston R, Ionica M, Papi A, Pignatel GU, Haino S (2008). Thermal and Electrical Characterization of Silicon Photomultiplier. IEEE Trans. Nucl. Sci.

[38] Tur C, Solovyev V, Flamanc J (2010). Temperature characterization of scintillation detectors using solid-state photomultipliers for radiation monitoring applications. Nucl. Instr. Meth. Phys. Res. A.

[39] Du Y, Retiere F (2008). After-pulsing and cross-talk in multi-pixel photon counters. Nucl. Instr. Meth. Phys. Res. A.

[40] Otte A (2009). On the efficiency of photon emission during electrical breakdown in silicon. Nucl. Instr. Meth. Phys. Res. A.

[41] Ninkovic J, Hartmann R, Holl P, Lutz G, Merck C, Mizroyan R, Hans-Günther M, Otte A-N, Richter R, Soltau H, Teshima M (2007). The Avalanche drift diode-A backilluminated Silicon Photomultiplier. Nucl. Instr. Meth. Phys. Res. A.

[42] Piemonte C (2006). A new Silicon Photomultiplier structure for blue light detection. Nucl. Instr. Meth. Phys. Res. A.

[43] Ninkovic J, Andricek L, Liemann G, Lutz G, Hans-Günther M, Richter R, Schopper F (2010). SiMPI– An avalanche diode array with bulk integrated quench resistors for single photon detection. Nucl. Instr. Meth. Phys. Res. A.

[44] Buzhan P, Dolgoshein B, Ilyin A, Kaplin V, Klemin S, Mizroyan R, Popova E, Teshima M (2009). The cross-talk problem in SiPMs and their use as light sensors for imaging atmospheric Cherenkov telescopes. Nucl. Instr. Meth. Phys. Res. A.

[45] Llosa G, Battiston R, Belcari N, Boscardin M, Collazuol G, Corsi F, Dalla Betta G-F, Del Guerra A, DInu N, Levi G, Marcatili S, Moehrs S, Marzocca C, Piemonte C, Pozza A (2008). Novel Silicon Photomultipliers for PET Applications. IEEE Trans. Nucl. Sci.

[46] Surti S, Kuhn A, Werner ME, Perkins AE, Kolthammer J, Karp JS (2007). Performance of Philips Gemini TF PET/CT Scanner with Special Consideration for Its Time-of-Flight Imaging Capabilities. J. Nucl. Med.

[47] Jakoby B, Bercier Y, Conti M, Casey M, Gremillion T, Hayden C, Bendriem B, Townsend DW Performance Investigation of a Time-of-Flight PET/CT Scanner.

[48] Wilson J, Turkington T TOF-PET Small-Lession Image Quality Measured Over a Range of Phantom Sizes.

[49] Degenhardt C, Prescher G, Frach T, Thon A, de Gruyter R, Schmitz A, Ballizany R The Digital Silicon Photomultiplier–A Novel sensor for the Detection of Scintillation Light.

[50] Frach T, Prescher G, Degenhardt C, de Gruyter R, Schmitz A, Ballizany R The Digital Silicon Photomultiplier–Principle of Operation and Intrinsic Detector Performance.

[51] Niclass C, Favi C, Kluter T, Monnier F, Charbon E (2009). Single-photon synchronous detection. IEEE J. Solid-State Circuits.

[52] Linga K, Godik E, Seemungal W, Shushakov D, Shubin VE (2005). Ultra low noise photodetectors with internal discrete amplification. Proc. SPIE.

[53] Linga K, Godik E, Krutovl J, Shushakov D, Shubin VE, Vinogradov SL, Levin EV (2006). Solid State Photomiltiplier: Noise Parameters of Photodetectors with Internal Discrete Amplification. Proc SPIE.

